# Supplementary material for: Visualizable detection of nanoscale objects using anti-symmetric excitation and non-resonance amplification
Source: Nat Commun. 2020 Jun 2;11:2754. doi: 10.1038/s41467-020-16610-0 (PMC7265281; doi:10.1038/s41467-020-16610-0)
Supplement: Supplementary file 1 — Supplementary Information [file 41467_2020_16610_MOESM1_ESM.pdf]

Visualizable Detection of Nanoscale Objects Using Anti-Symmetric Excitation and Non-Resonance Amplification

Zhu *et al.*

Supplementary Note 1: Mathematical model for generating an electromagnetic canyon

The pair of nanowires and the object form an electrostatic ensemble whose modes can be found by first solving a self-sustained eigenvalue problem for each individual nanostructure[1]

$$\sigma_i(\mathbf{r}) = \frac{\gamma_i}{2\pi}\oint \sigma(\mathbf{r}_i)\frac{(\mathbf{r}-\mathbf{r}_i)\cdot\mathbf{n}_i}{\left|\mathbf{r}-\mathbf{r}_i\right|^3}\mathrm{d}S_i \quad \text{and}$$
$$\tau_i(\mathbf{r}) = \frac{\gamma_i}{2\pi}\oint \tau(\mathbf{r}_i)\frac{(\mathbf{r}_i-\mathbf{r})\cdot\mathbf{n}_i}{\left|\mathbf{r}-\mathbf{r}_i\right|^3}\mathrm{d}S_i \quad (i=1,2,3), \qquad \text{(Supplementary Equations 1)}$$

where σ_i, τ_i, and $\mathbf{n}_i$ are the charge, dipole, and normal vector at position $\mathbf{r}$ on the surface of the ith nanostructure. $\gamma_i = (\varepsilon_i - \varepsilon_{\text{eff}\_i}) / (\varepsilon_i + \varepsilon_{\text{eff}\_i})$ is the eigenvalue associated with the corresponding eigenvalue equation. ε_i is the electric permittivity of the ith nanostructure, and $\varepsilon_{\text{eff}\_i}$ is the effective background permittivity obtained by considering the substrate-induced image nanostructure.[2] Once the materials and dimensions are given, the surface charge eigenmodes $\sigma_i^k(\mathbf{r})$ for $k=\{1,2,\ldots,\infty\}$ can be uniquely determined and the surface charge distribution can be represented by a superposition of these eigenmodes. Note that the eigenfunctions describe the intrinsic properties of a self-sustained system. Thus, they are valid irrespective of the external illumination source used and are only determined by the materials and topology. The electric field at an arbitrarily spatial position $\mathbf{r}$ from the ensemble is given by

$$\mathbf{E}(\mathbf{r}) = \sum_{i=1}^{M}\oint \mathbf{g}(\mathbf{r}-\mathbf{r}_i)\sum_{k_i=1}^{\infty} c_i^{k_i}\sigma_i^{k_i}\mathrm{d}S_i \quad (i=1,2,3), \qquad \text{(Supplementary Equations 2)}$$

where $\mathbf{g}(\mathbf{r}-\mathbf{r}_i)$ is the vectorial Green's function, and $c_i^{k_i}$ is the k_i^{th} coefficient to be determined for the k_i^{th} eigenmode $\sigma_i^{k_i}$ for the ith nanostructure. M denotes the number of isolated nanostructures in the ensemble. Here $M = 3$. The excitation of these modes in a nanostructure is determined by both the external sources and the evanescent fields due to the nearby nanostructures. It is therefore

the undetermined coefficients in the eigenmode expansion for the surface charge and for the surface dipole distribution that include the effects from the self-coupling among the nanostructures of the ensemble. The expansion coefficient $c_i^{k_i}$ can be described via the biorthogonality between $\sigma_i^{k_i}$ and $\tau_i^{k_i}$ [2]

$$c_i^{k_i} = \beta_i^{k_i} \oint \tau_i^{k_i} \mathbf{n}_i \cdot \left[\mathbf{E}_{i0} + \sum_{p=1}^{M} \mathbf{E}_{ip} \right] \mathrm{d}S_i, \qquad \text{(Supplementary Equations 3)}$$

where $\beta_i^{k_i} = \frac{\left(\varepsilon_i - \varepsilon_{\mathrm{eff}\_i}\right)\left(\varepsilon_i^{k_i} - \varepsilon_{\mathrm{eff}\_i}\right)}{\varepsilon_i^{k_i} - \varepsilon_i}$ and $\varepsilon_i^{k_i}$ is the real permittivity associated with the k_ith eigenvalue of the ith nanostructure. $\mathbf{E}_{i0}$ and $\mathbf{E}_{ip}$ respectively denote the applied field and the electric field arising from the pth nanostructure at position $\mathbf{r}_i$ on the surface of the ith nanostructure. Here, the electric field from the ith nanostructure acting on itself is assumed to be zero. If the observation point $\mathbf{r}$ in Supplementary Equation 2 is on the surface of the jth nanostructure, the expansion coefficient can be obtained by combining Supplementary Equations 2 and 3 followed by algebraic operations

$$c_i^{k_i} = \xi_{i0}^{k_i} + \sum_{h=1}^{\infty} \sum_{l=1}^{M} \sum_{m=1}^{M} \sum_{d=1}^{\infty} f_{ilm}^{k_i hd} \xi_{m0}^{d}, \qquad \text{(Supplementary Equations 4)}$$

where $f_{ilm}^{k_i hd} = \left(\delta^{k_i h} \delta_{il} - C_{li}^{hk_i}\right)^{-1} C_{lm}^{hd}$ and δ denotes the Kronecker delta function. $\xi_{i0}^{k_i}$ and ξ_{m0}^{d} are given by

$$\begin{aligned} \xi_{i0}^{k_i} &= \beta_i^{k_i} \oint \tau_i^{k_i}(\mathbf{r}_i)\mathbf{n}_i \cdot \mathbf{E}_{i0} \mathrm{d}S_i \text{ and} \\ \xi_{m0}^{d} &= \beta_m^{d} \oint \tau_m^{d}(\mathbf{r}_m)\mathbf{n}_m \cdot \mathbf{E}_{m0} \mathrm{d}S_m, \end{aligned} \qquad \text{(Supplementary Equations 5)}$$

where $\mathbf{E}_{i0}$ and $\mathbf{E}_{m0}$ denote the applied field on the surfaces of the ith and mth nanostructures, respectively. Here, we should mention that the subscripts i and m do not have any essential

difference; the only difference is that i denotes the quantity to be determined, while m denotes the quantity to be superposed in Supplementary Equation 4 arising from the self-coupling within the ensemble. $C_{li}^{hk_i}$ is the term that describes the coupling from the ith particle to the lth particle

$$C_{li}^{hk_i} = \beta_l^h \oint \oint \tau_l^h(\mathbf{r}_l)\mathbf{n}_l \cdot \mathbf{g}(\mathbf{r}_l - \mathbf{r}_i)\sigma_i^{k_i}(\mathbf{r}_i)\mathrm{d}S_l \mathrm{d}S_i. \qquad \text{(Supplementary Equations 6)}$$

C_{lm}^{hd} can be derived analogously by simply updating the subscripts and superscripts. If the bright modes can be excited via external sources,[3] the nanostructure radiates out propagation waves into the surrounding background and thus the effect of radiation damping should be considered in the electrostatic interaction. For deep sub-wavelength objects, dipolar radiation dominates the oscillation damping; thus, higher-order multipoles can be neglected. Accordingly, the expansion coefficient in Supplementary Equation 4 can be modified by adding the damping terms[3]

$$c_i^{k_i} = \xi_{i0}^{k_i} + j\alpha_i P_i^{k_i} + \sum_{h=1}^{\infty}\sum_{l=1}^{M}\sum_{m=1}^{M}\sum_{d=1}^{\infty} f_{ilm}^{k_i hd}\left(\xi_{m0}^{d} + j\alpha_m P_m^{d}\right), \qquad \text{(Supplementary Equations 7)}$$

where $\alpha_i = k^3 / 6\pi\varepsilon_{\text{eff\_}i}$ and $\alpha_m = k^3 / 6\pi\varepsilon_{\text{eff\_}m}$. The dipole term $P_i^{k_i}$ is given by

$$P_i^{k_i} = \beta_i^{k_i} \oint \tau_i^{k_i}(\mathbf{r}_i)\mathbf{n}_i \cdot \mathbf{p}_i \mathrm{d}S_i, \qquad \text{(Supplementary Equations 8)}$$

where $\mathbf{p}_i$ is the dipole moment of the ith nanostructure. P_m^d is given by Supplementary Equation 8 with an updated superscript. Knowing the expansion coefficients, the electric field at an arbitrary position $\mathbf{r}$ can be computed via Supplementary Equation 2 provided the eigenfunctions are predetermined.

As shown in Fig. 2, the left and right nanowires are identical in both their material and their topology; thus, the corresponding eigenfunctions are normally identical, i.e., $\sigma_1^{k_i} = \sigma_2^{k_i}$ and $\tau_1^{k_i} = \tau_2^{k_i}$. Accordingly, the elements of the coupling matrix described in Supplementary Equation 6, would

normally satisfy $C_{12}^{hk_i} = C_{21}^{hk_i}$ and $C_{11}^{hk_i} = C_{22}^{hk_i}$. Here, we should recall the fact that the coupling matrices do not depend on the external sources. In conventional excitation where the transverse area of the nanostructures is much smaller than the beam size, the local gradient of the electric field can be neglected. Thus, the field that excites the left and right nanowires can be regarded as identical to each other, i.e., $\mathbf{E}_{10} = \mathbf{E}_{20}$; see Fig. 2a. This indicates that $\xi_{10}^d = \xi_{20}^d$. Due to the geometrical symmetry without the nanoscale object, $P_1^{k_i} = P_2^{k_i}$ because of $\mathbf{p}_1 = \mathbf{p}_2$. Hence, we have $c_1^{k_i} = c_2^{k_i}$ and the electric field at an arbitrary point on the symmetric plane (SP) from the pair of nanowires is always the result of constructive interference. When the object is present, the symmetry is broken, and the elements of coupling matrix are changed by the coupling between the object and the pair of nanowires, i.e., $c_i^{k_i}\Big|_{\text{pert}} = c_i^{k_i} + \Delta c_i^{k_i}\Big|_{\text{pert}}$ $(i = 1, 2)$, where $\Delta c_i^{k_i}\Big|_{\text{pert}}$ denotes the change and is given by

$$
\begin{aligned}
\Delta c_i^{k_i}\Big|_{\text{pert}} &= j\alpha_i \left(P_i^{k_i}\Big|_{\text{pert}} - P_i^{k_i} \right) + \sum_{h=1}^{\infty}\sum_{l=1}^{3}\sum_{m=1}^{3}\sum_{d=1}^{\infty} f_{ilm}^{k_i hd} \left(\xi_{m0}^d + j\alpha_m P_m^d\Big|_{\text{pert}} \right) \\
&\quad - \sum_{h=1}^{\infty}\sum_{l=1}^{2}\sum_{m=1}^{2}\sum_{d=1}^{\infty} f_{ilm}^{k_i hd} \left(\xi_{m0}^d + j\alpha_m P_m^d \right) \quad (i = 1, 2),
\end{aligned}
$$

(Supplementary Equations 9)

where $P_m^d\Big|_{\text{pert}}$ denotes the perturbed dipole distribution. The electric field on the SP then can be represented as

$$
\mathbf{E}_{\text{SP}}(\mathbf{r}) = \mathbf{E}_{\text{SP}}(\mathbf{r}, c_1^{k_i}) + \mathbf{E}_{\text{SP}}(\mathbf{r}, c_2^{k_i}) + \mathbf{E}_{\text{SP}}(\mathbf{r}, \Delta c_1^{k_i}\Big|_{\text{pert}}) + \mathbf{E}_{\text{SP}}(\mathbf{r}, \Delta c_2^{k_i}\Big|_{\text{pert}}) + \mathbf{E}_{\text{SP}}(\mathbf{r}, c_3^u),
$$

(Supplementary Equations 10)

where c_3^u is the coupling matrix with respect to the object. Each term in the right-hand side of Supplementary Equation 10 has an explicit expression that can be easily obtained by substituting

$c_i^{k_i}\Big|_{\text{pert}} = c_i^{k_i} + \Delta c_i^{k_i}\Big|_{\text{pert}}$ $(i = 1, 2)$ followed by an expansion according to the serial number of particles. From the previous discussion, we recall that $\mathbf{E}_{\text{SP}}(\mathbf{r}, c_1^{k_i})$ and $\mathbf{E}_{\text{SP}}(\mathbf{r}, c_2^{k_i})$ do not cancel out but instead form a strong constructive interference on the SP. If the dimension of the object is much smaller than that of the nanowires, the terms $[\mathbf{E}_{\text{SP}}(\mathbf{r}, \Delta c_1^{k_i}\Big|_{\text{pert}}) + \mathbf{E}_{\text{SP}}(\mathbf{r}, \Delta c_2^{k_i}\Big|_{\text{pert}}) + \mathbf{E}_{\text{SP}}(\mathbf{r}, c_3^{u})]$ related to the object can be overwhelmed by the constructive interference. Hence, it is difficult to directly observe the nanoscale objects from the far-field images, which is especially the case when the pair of nanowires has a sub-wavelength gap. If we instead excite the nanowires into an anti-symmetric state, i.e., $\mathbf{E}_{20} = -\mathbf{E}_{10}$, we have $\xi_{10}^{k_i} = -\xi_{20}^{k_i}$, $\xi_{10}^{d} = -\xi_{20}^{d}$, $P_1^{k_i} = -P_2^{k_i}$, and $P_1^{d} = -P_2^{d}$. This indicates $c_1^{k_i} = -c_2^{k_i}$ and thus the unperturbed terms $\mathbf{E}_{\text{SP}}(\mathbf{r}, c_1^{k_i})$ and $\mathbf{E}_{\text{SP}}(\mathbf{r}, c_2^{k_i})$ cancel out each other on the SP in the near-field region, leaving only the object-related terms. This means we may be able to observe the nanoscale object even if the investigated object is much smaller than the wavelength.

Supplementary Note 2: The role of symmetry in generating an electromagnetic canyon—dipolar approximation

Consider a pair of closely positioned identical dielectric nanoscale objects on the x-axis at xyz coordinates ($-p$, 0, 0) and (p, 0, 0), where $p \ll \lambda$. Assume that they are illuminated by a monochromatic beam propagating along the z-direction in an epi-illumination microscope. Typically, in classical imaging, the excitation is described as beam-like, i.e., the field has a constant or a slowly-varying cross-section, e.g., a plane wave or a Gaussian beam, respectively. When the objects are located in the path of a collimated beam (widefield imaging) or at the beam's focus (confocal imaging), they are simultaneously and isotropically excited and form the symmetric

polarization state. This is equivalent to two in-phase electric dipoles of equal amplitude; see Supplementary Figure 1a. In contrast, consider anisotropically exciting the objects and forming the anti-symmetric polarization state. The two dipoles now have equal amplitudes but oscillate perfectly out-of-phase. Supplementary Figure 1b vividly depicts this using two arrows with opposite orientations. Using the electric dipole approximation and starting from equation 9-18 of Ref. 4, we can express the complex amplitude $\mathbf{E}_t(\mathbf{r})$ at an arbitrary observation point $\mathbf{r}$ in both the symmetric and anti-symmetric cases as:

$$\mathbf{E}_t(\mathbf{r}) = \xi_1\mathbf{D}_1 + \xi_2\mathbf{D}_2 + \gamma_1\left(\mathbf{D}_1\cdot\hat{\mathbf{r}}_1\right)\hat{\mathbf{r}}_1 + \gamma_2\left(\mathbf{D}_2\cdot\hat{\mathbf{r}}_2\right)\hat{\mathbf{r}}_2, \qquad \text{(Supplementary Equations 11)}$$

where $\mathbf{D}_1$ and $\mathbf{D}_2$ are the complex amplitudes of the dipoles, $\mathbf{D}_1(t)$ = Re[$\mathbf{D}_1 e^{-i\omega t}$] and $\mathbf{D}_2(t)$ = Re[$\mathbf{D}_2 e^{-i\omega t}$]. $\hat{\mathbf{r}}_1$ and $\hat{\mathbf{r}}_2$ are the unit vectors originating from the left and right dipoles, respectively, and going to the point $\mathbf{r}$. ξ_j and γ_j (j = 1, 2) are complex coefficients that depend on the distance between the observation point and the source, i.e.,

$$\begin{aligned}
\xi_j &= \frac{k^2 e^{ikr_j}}{4\pi\varepsilon_0 r_j}\left[1 - \frac{1}{k^2 r_j^2} + \frac{i}{kr_j}\right] \\
\gamma_j &= \frac{k^2 e^{ikr_j}}{4\pi\varepsilon_0 r_j}\left[\frac{3}{k^2 r_j^2} - \frac{3i}{kr_j} - 1\right], \ j = 1, 2.
\end{aligned} \qquad \text{(Supplementary Equations 12)}$$

Here, k is the wavenumber, ε_0 is the permittivity of vacuum, and r_j denotes the distance from the jth dipole to the arbitrary observation point. The first two and last two terms on the right-hand side of Supplementary Equation 11 can be interpreted as the dipole-induced and the position offset-induced field contributions, respectively, i.e., $\mathbf{E}_d = \xi_1\mathbf{D}_1 + \xi_2\mathbf{D}_2$ and $\mathbf{E}_r = \gamma_1\left(\mathbf{D}_1\cdot\hat{\mathbf{r}}_1\right)\hat{\mathbf{r}}_1 + \gamma_2\left(\mathbf{D}_2\cdot\hat{\mathbf{r}}_2\right)\hat{\mathbf{r}}_2$. We denote the phase difference between $\mathbf{D}_1$ and $\mathbf{D}_2$ as α, where

$0 \leqslant \alpha \leqslant \pi$. Thus, α equals 0 and π for the symmetric and anti-symmetric cases shown in Supplementary Figures 1a and 1b, respectively.

The symmetry properties of the x, y, and z electric field components in Supplementary Equation 11 across the $x = 0$ and the $y = 0$ planes are the key factors in determining whether the electromagnetic canyon (EC) can be generated. Moreover, α determines these symmetry properties. The symmetric state results in constructive interference and a merging of the objects in the image while the anti-symmetric state creates a destructive interference splitting-line (i.e., the EC) across which we can perfectly resolve the two objects. Using the analytic expression in Supplementary Equation 11, we summarize the symmetry properties of each field component for different dipole excitations in Supplementary Table 1. To start the analysis, let us compare symmetric and anti-symmetric y-polarized dipoles. For these cases, E_y is the dominant near-field component; see Supplementary Figure 2. For symmetric y-polarized dipoles, E_y is symmetric across both planes and thus it is not possible to resolve the dipoles in the microscope image; see Supplementary Figure 1c. The same conclusions hold for symmetric x-polarized and z-polarized dipoles (not shown). However, for anti-symmetric y-polarized dipoles, E_y is anti-symmetric in x. Thus, there is a near-field splitting line for E_y and the objects are resolved in the microscope image; see Supplementary Figure 1j. Moreover, E_x is anti-symmetric in y while E_z is anti-symmetric in both x and y. Anti-symmetry across at least one plane for each near-field component produces a perfect destructive interference in the microscope image at the intersection of the two planes. Thus, the microscope image will have zero intensity at the center point $(x, y) = (0, 0)$. See Supplementary Figure 3a, which shows the two dipoles are resolved with infinite contrast ratio. Contrast ratio is defined as the ratio of the peak intensity in the image to the value at (0, 0). A paradigm-shifting consequence of the anti-symmetry in the excited dipole moments is an EC with perfect null.

Diffraction and interference in the lens-based system now play the role of catalyst instead of barrier for creating high contrast ratio images. Note that we assumed that the centroid of the nanowire pair is located on the optical axis of the microscope so that the system respects field symmetries. Under this assumption, a perfect EC is formed, regardless of the NA of the objective or the spacing of the nanowires. This is not a limiting assumption because in practice, even when the nanowire centroid is off-axis, the gap spacing is small enough that the radiation pattern from each nanowire is equally collected by the objective. For anti-symmetric x-polarized and z-polarized dipoles, we also obtain a splitting line and anti-symmetry across $x = 0$ for the near-field maps of E_x and E_z, respectively. See Supplementary Table 1. However, the symmetry across both planes of E_z for x-polarized dipoles and of E_x for z-polarized dipoles causes the contrast ratio to decrease to approximately 10 (a slightly imperfect but usable EC) and 1 (no EC), respectively; see Supplementary Figures 3b and c. Whereas z-polarized dipoles do not form an EC, the x-polarized dipoles still form the canyon because the near-field longitudinal component E_z has a weaker effect on the far-field microscope image than the near-field transverse components, E_x and E_y. To generate an EC, α need not be exactly 180°, i.e., tolerance exists. We can excite the objects into a partially anti-symmetric state. See Supplementary Figures 1d-i, where we can clearly see an EC for two objects spaced by $d = 2p = \lambda/4$ when $\alpha \geq 150°$. We herein define a gap-dependent threshold $\alpha_t(d)$ to be the smallest value of α for which the two objects form an EC, i.e., a local minima exists in the middle of the two peaks. Supplementary Figure 1 shows that $\alpha_t(\lambda/4) = 150°$. As d is reduced, the value of $\alpha_t(d)$ approaches 180°.

Under anti-symmetric excitation, not only can we generate the EC, but we can observe this EC using a microscope objective with an arbitrarily small NA; compare Supplementary Figure 4b with Supplementary Figure 4a. This is because the microscope objective's NA does not change

the symmetry properties of the fields. For both the x-polarized and y-polarized anti-symmetric cases, when the gap, $2p$, is reduced below Abbe's limit, the peak intensity begins to drop off rapidly (approximately as p^2), but we should mention that the dropped intensity belongs to the pair of objects (nanowires in Fig. 2), not to an object (if present). However, the apparent object gap (AOG), i.e., the gap in the image space divided by the system magnification, converges to a constant after the expected decrease in the regime of geometrical optics. See Supplementary Figures 4c and 4d. The overlap of the curves for x- and y-polarization indicates that an arbitrary linear combination of the transversally anti-symmetric polarization does not significantly alter the formation of the EC for the two objects; see Supplementary Figures 4e and 4f. If $\mathbf{D}_1$ and $\mathbf{D}_2$ include anti-symmetric z-components, the microscope image degenerates, but there is still a local minimum (i.e., an EC is formed) if the magnitude of the longitudinal component of the dipole moments does not exceed three times that of the transverse component; see Supplementary Figure 5. The above results and conclusions directly extend to polychromatic (i.e., white-light) excitation that is anti-symmetric for the dominant wavelengths, which builds a solid foundation for experimental validations. Note that the amplitude and absolute phase of each wavelength can be arbitrary.

Supplementary Note 3: Details of the imaging setup in simulations

The coherent optical imaging microscope used in the simulation for Figs. 2 and 3 is in a widefield configuration with a 100× magnification and a 0.8 input numerical aperture (NA) of the objective. The output NA of the imaging optics is chosen as 1. To compute the vectorial images of nanostructures, three procedures, i.e., the definition of sources, the computation of near-field, and the propagation of EM field for imaging, are implemented. Here, we should mention that any other

input NA (for example, NA = 0.4 in the manuscript) can be chosen for the following analysis without affecting the anti-symmetric conclusions.

3.1 Definition of sources and near-field computation

The near-field of the nanostructure assembly used to compute the images are obtained via the finite-different time-domain method. For a pair of nanowires positioned on top of a substrate, in order to anti-symmetrically excite them, we use the standing waves formed by two-beam interference with an inclined angle of 60º, as proposed in Fig. 2c. However, we should remind our readers that two-beam interference is not the only way to achieve anti-symmetric excitation; any vectorial beam that has a feature of a local π-shift in phase can be utilized for anti-symmetric excitation, provided the pair of objects is positioned symmetrically about the null. A near-field observation plane that is above the investigated area is used to record the scattering field from the excited nanostructure assemble. The area of the observation plane should be large enough to allow most of the time-averaged backward scattering power (>95%) to flow through.

3.2 Near-field decomposition and imaging

The electric field captured by the observation plane is decomposed into a series of plane waves using far-field projection, after which the plane wave components within the input NA are focused onto the image plane by chirped z-transform.[5] This imaging methodology is naturally succinct and fast. To evaluate its accuracy, we applied another imaging method that is referred to as the equivalent magnetic-dipole (EMD) based vectorial electromagnetic field imaging.[6-8] In this method, the electric field on the observation plane is decomposed into many EMDs followed by the ray tracing for all the field lines of EMDs within the input NA using the generalized Jones matrix formalism. The Debye-Wolf integral then can be applied to calculate the image of all the EMDs. If the observation plane is large enough [to allow most of the time-averaged backward

scattering power (>95%) to flow through] and if the sampling interval for EMDs is small enough ($\lambda/3$ in our estimation), the EMD based method can give an accurate image for an arbitrary target. The drawback of the EMD based method is that it is time-consuming if the number of EMDs is very large. For the computation of the image with 150 × 150 observation points corresponding to 301 × 301 EMDs, it takes 13.3 minutes in MATLAB integrated programming environment on an in-house built workstation with two Intel Xeon E5-2683 v3 2.0 GHz 28-core processors. However, by parallelizing the program onto 16 Pacini computing nodes of Cisco's Arcetri cluster using C++, the computation time reduced to only 8.4 s. As the EMD based method naturally inherits the vectorial properties of EM imaging, it is a reasonable benchmark to estimate the chirped z-transform based method. Accordingly, we compute the microscope images on the best focal plane for a dipole pair (with $\lambda/15$ gap) that is polarized along y-direction with anti-symmetry about x = 0; see Supplementary Figure 6. The intensity curves corresponding to the central cross-sections of the two images from both methods highly overlap with only a minor mismatch on the sidelobes. Hence, the time-efficient chirped z-transform based imaging method is accurate enough to give reasonable images for the investigated samples under the current widefield configuration.

3.3 Localized illumination to minimize the boundary effect and background reflection

For a given computational domain, the application of global illumination can result in boundary effects and background reflection, which affect the fidelity of the optical image. To explain this issue, we use schematics shown in Supplementary Figure 7 and discuss the importance of localized illumination. As shown in Supplementary Figure 7a, global illumination (green arrows) produces both the scattering field from the nanostructure (blue arrows) and the background reflection from the extended substrate (lilac arrows). These will be recorded in the observation plane (purple line). Here, we should emphasize again that the observation plane is not a real plane but rather a monitor

that records the EM field for the computation of optical images. Because of finite computational resources, we need to truncate the computation domain; see the dashed black lines marked with "boundary." The global illumination will result in significant boundary scattering/reflection artifacts, which generate a harmful scattering field in the record plane, which is referred to as the boundary effect; see the schematic curves plotted on the observation plane. The boundary effect will dominate the scattering field from objects and distort the optical images, especially considering the fact the scattering of the object is weak. To address this issue, we can introduce the localized illumination, i.e., we introduce an illumination box in which only the surrounded nanostructure (consisting of the nanowire pair and the object) and a small part of the substrate are excited (see the dashed yellow box in Supplementary Figure 7b). Because there is no excitation outside the box, the boundary effect and background reflection can be minimized. This method enables a high-fidelity computation for the EM field of nanostructures in the imaging plane.

Supplementary Note 4: Generation of electromagnetic canyons with various objects

We now consider the generation of ECs with various real objects, where the finite dimension-induced non-uniform polarization and influence of the background cannot be neglected. See Supplementary Figure 8. The mathematical model developed in Supplementary Note 1 indicates that any pair of objects can be utilized to generate the EC. The first object type is a silicon bowtie on top of a SiO_2 substrate. The gap size (20 nm) is smaller than many common bowtie structures.[9,10] The left and right triangles are impinged by a y-polarized transverse beam with phases $-\pi/2$ and $\pi/2$, respectively. Each beam for anti-symmetric excitation is a localized plane wave (see the inset at the top left corner in Supplementary Figure 8e). This is a very simple and general method to excite the anti-symmetric state in simulation. The brightfield microscope captures images of the bowtie area in epi-illumination mode. Supplementary Figure 8a shows that

the peaks for the left and right triangles are clearly resolved and that an EC has been generated in between. The SiO_2 substrate, which essentially plays the role of a mirror bowtie, does not alter the anisotropic polarization but only changes the strength of electric polarization. In transmission mode, the background behaves as the mirror image of the object. Supplementary Figure 8b shows the transmission image for nanoholes etched on an thin plate of infinite area. Researchers often use the double-nanowire structure as the gold standard for evaluating a microscope's resolving power. Supplementary Figures 8c and d show clear ECs for epi-illumination and transmission modes, respectively. To further understand tolerances, we separately explored the effects of reducing the excitation amplitude on the left side and the effect of adding line edge roughness (LER) to the right-side of the double-nanowire structure. Supplementary Figures 8e and f show that breaking the perfectly anti-symmetric configuration by introducing either excitation asymmetry or roughness, respectively, indeed disturbs the images. The result is an asymmetric pattern in the image space, but still the ECs are clearly generated even when the amplitude of the left excitation is only 40% of that of the right excitation or when a $\lambda/15.7$-scale LER exists.

Supplementary Note 5: Experimental Setup and Sample

Supplementary Figure 9 shows photographs of the experimental setup, which consists of both fiber optic and free space elements. Supplementary Figure 10 illustrates the process to align and calibrate the setup. Additional details can be found in the Methods section.

Supplementary Figure 11a shows a full-field SEM image of the fabricated sample. The three large rings are used for orienting the wafer and for performing coarse rotational and translational alignment of the wafer under the microscope. Supplementary File 1 is an archive containing a Graphic Database System (GDS) file that provides the layout of the sample patterns in units of microns and a Portable Document Format (PDF) showing the layout at 1000x

magnification. All patterns include a -20 nm bias on all sides relative to the nominal design dimensions to compensate for blurring from the non-zero beam size of electron beam lithography thereby enabling the fabricated dimensions to more closely match the design dimensions. The total exposure field size is 2.533 mm wide by 1.114 mm tall. In addition to the three large rings, the layout consists of 44 die of nanowire devices that are arranged in 5 rows and 11 columns spaced by 240 µm in each direction. Each die has 6 large triangles on the bottom and 7 large triangles on the left to simplify the process of locating each of the 42 individual nanowire devices within the die from a large field of view optical image. In each die, half of the devices are oriented along the *x*-direction and half along the *y*-direction to enable excitation of both *x*-polarized and *y*-polarized dipoles with a single illumination configuration. The bottom row of die consist of double-nanowire devices with gaps (G_1) ranging from 0 nm to 1000 nm in increments of 50 nm. From left to right, the first 4 columns have a nominal width of 50 nm (width of digitized pattern is 10 nm) and nominal lengths of 100 nm, 500 nm, 1 µm, and 2 µm, respectively, while the last 4 columns have a nominal width of 100 nm and these same nominal lengths. The middle three rows of die consist of quad-nanowire devices with the same set of G_1 gaps as the double-nanowire devices in each die. Each nanowire segment has a fixed nominal width of 100 nm and length of 7 µm. From left to right, each column of die has a different gap G_2 ranging from 0 to 500 nm in increments of 50 nm to make a quad-nanowire pattern. As a clarifying example, when $G_2 = 0$, the pattern is a double-nanowire structure with gap G_1 and 14-µm nominal length but when $G_1 > 0$ and $G_2 > 0$, the pattern is a quad-nanowire structure with 7-µm long nanowires. The second row from the bottom has no defects. The third row, labeled with letter 'X' in the bottom left corner of each die, has a parallel bridge (BX) defect that joins two of the four nanowires across the G_2 gap. The fourth row from the bottom, labeled with letter 'Y', has a perpendicular bridge (BY) defect that joins two of the

four nanowires across the G_1 gap. The fifth row, labeled with letter 'G' consists of ten-nanowire device structures with a fixed nominal width of 100 nm and length of 7 μm and periods ranging from 0 nm to 1000 nm in increments of 50 nm. All 3 of the die in this row are identical. Zoomed in images of devices from a few of the columns of the first three rows are presented in this paper. Supplementary Figures 11b and c show SEM images of typical patterns from the dies where the length of the nanowires are 2 μm and 14 μm, respectively.

Supplementary Note 6: Visualizable sensing of biological objects

Because of the small size and low refractive index contrast biological objects (e.g., viruses or molecule clusters) in an ambient environment such as air or water, proper choices for the nanowire material and the fabrication method must be made that considers both the signal level and the noise from fabrication imperfections including roughness. To mimic its size and index, we model an individual biological object as a square of SiO_2 material with variable size *w*. Supplementary Figure 12 shows the strength of the far-field signal versus the object size for different nanowire materials. The silicon nanowire pair has the strongest signal for the object and likewise would be the most sensitive to a fixed size fabrication imperfection. Conversely, the SiO_2 nanowire pair has the weakest signal and would be least sensitive to a fabrication imperfection. The material and fabrication method strongly influence the level of roughness. The roughness of surfaces and sidewalls is expected to be significantly lower in a crystalline material patterned with a high crystollographic selectivity atomic layer process than in an amorphous material or when patterned with a non-crystallographic method. Supplementary Figure 13 shows the

envisioned system for trapping and visualizing biological targets. The targets bind and thereby form perturbations to the uniform array of functionalized nanowires.

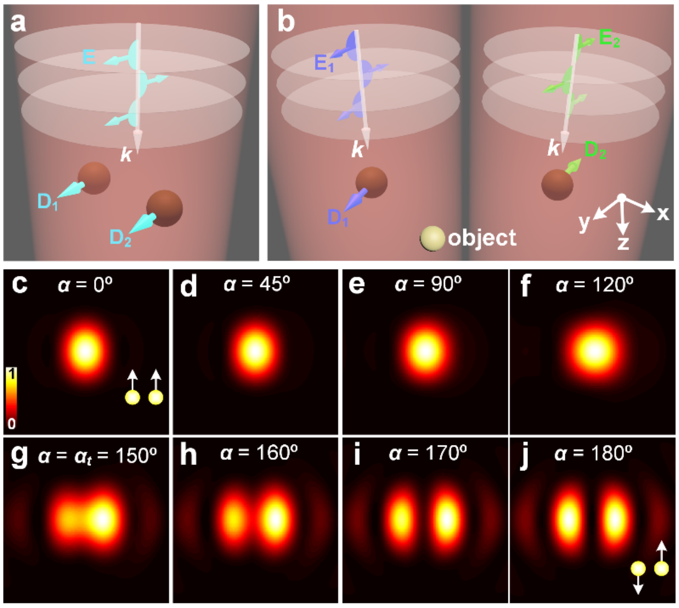

Supplementary Figure 1. **Comparison of symmetric and anti-symmetric excitation for creating an EC using a pair of point objects**. **a** and **b** Schematic showing symmetric and anti-symmetric illumination. The arrows on each object denote the excited dipolar moments. **c-j** Microscope intensity images of the pair of dipoles with a gap of $\lambda/4$ for various values of α. The insets on the bottom right corner of **c** and **j** denote the in-phase (symmetric) and out-of-phase (anti-symmetric) excitation states. The field of view is 1 μm × 1 μm in the sample space.

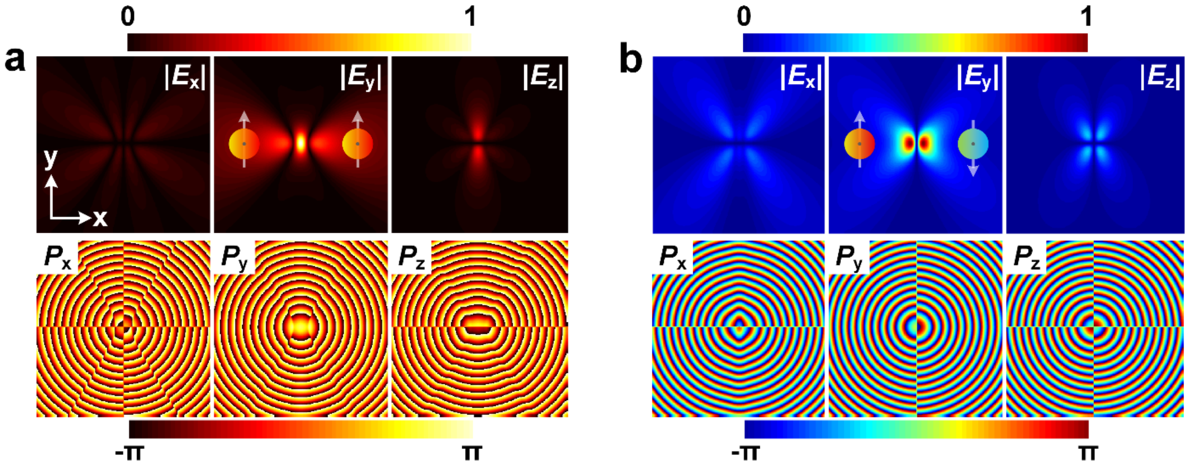

Supplementary Figure 2. Comparison of the near-fields for symmetric and anti-symmetric *y*-polarization. **a** and **b** Amplitude and phase distributions of different field components on a near-field plane that is 4-μm wide by 4-μm long and 1 μm above the *x*-*y* plane. The gap for the pair of dipoles is $\lambda/4$.

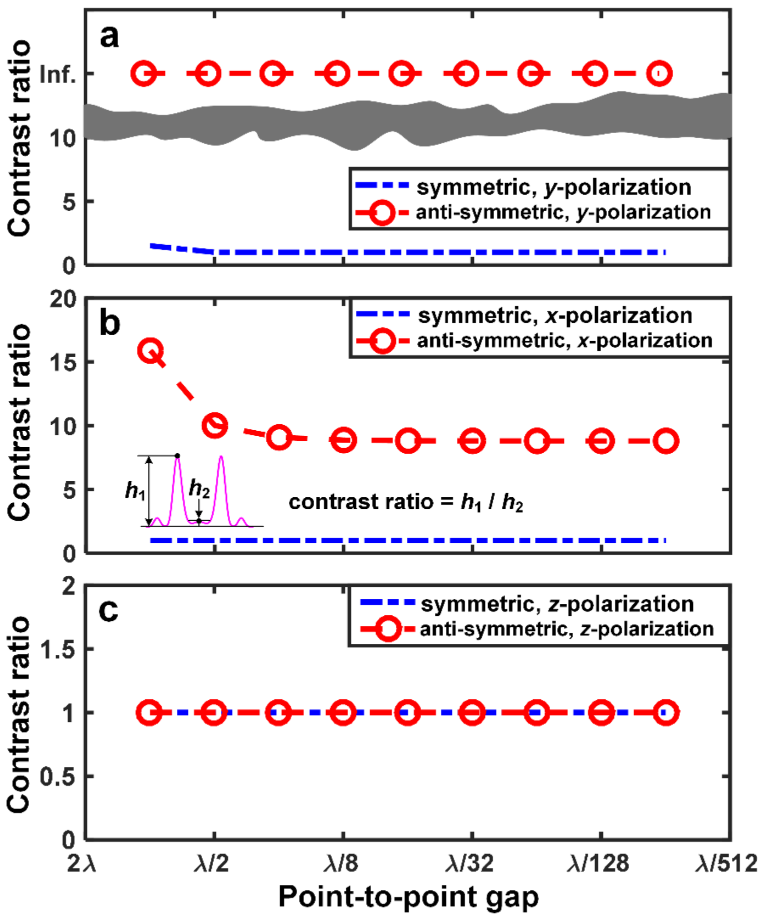

Supplementary Figure 3. **Contrast ratio as a function of the point-to-point gap**. **a** y-polarization. **b** x-polarization. **c** z-polarization. The red dashed and blue dotted curves denote the contrast ratio for the anti-symmetric and symmetric dipoles, respectively. The inset on the bottom left corner of **b** shows the definition of the contrast ratio, which is the ratio of the intensity at the brightest spot in the image to the intensity at the center of the image and is a measure of the quality of the EC.

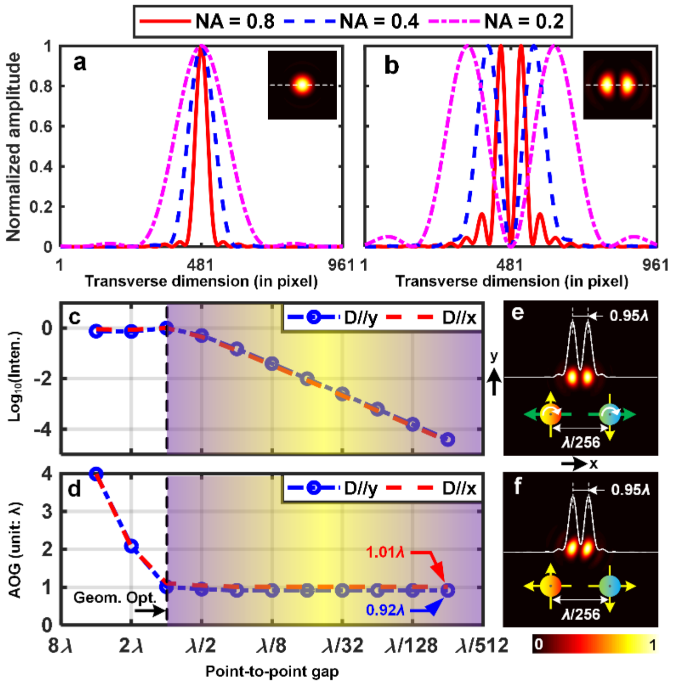

Supplementary Figure 4. **Characteristics of microscope images for dipoles with anti-symmetric transverse polarization**. **a** and **b** Effect of the NA of the objective lens on resolving power for a pair of *y*-polarized dipoles in the **a** symmetric and **b** anti-symmetric states. The gap for the pair of dipoles in **a** and **b** is $\lambda/4$ and the system magnification is fixed at 100×. The insets in **a** and **b** are the respective microscope images for NA = 0.2. **c** Peak intensity and **d** AOG curves as functions of the point-to-point gap for the anti-symmetric *x*-polarized and *y*-polarized dipoles. AOG is defined as the observed gap in the imaging space divided by the system magnification. **e** and **f** Microscope images of the anti-symmetric **e** right-circularly polarized and **f** $\pi/4$ linearly polarized dipoles.

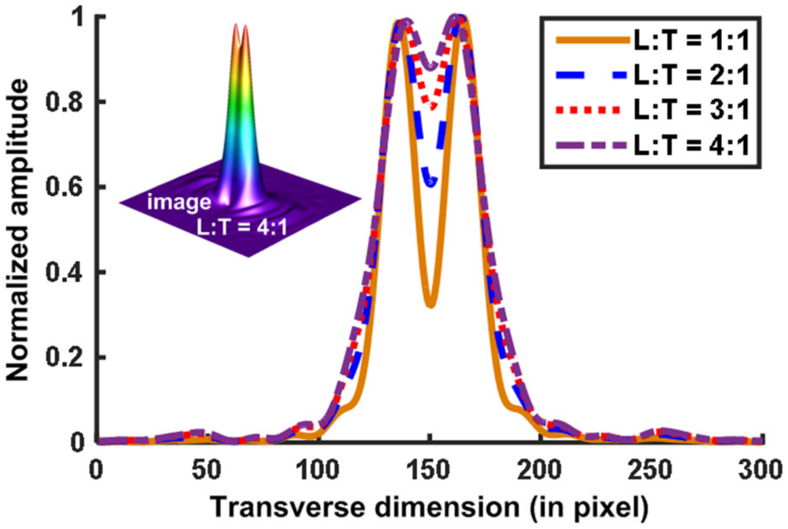

Supplementary Figure 5. **The detrimental effect of the longitudinal field component on the generation of the EC**. The gap for the pair of dipoles is $\lambda/4$. L: amplitude of the longitudinal component; T: amplitude of the transverse component. The magnification and input NA of the widefield microscope are 100× and 0.8, respectively. Each pixel on the microscope image is 2 µm × 2 µm, which corresponds to 20 nm × 20 nm in the sample space.

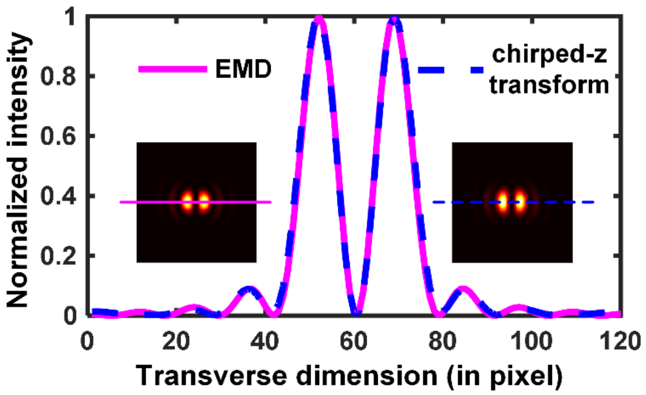

Supplementary Figure 6. **Validation for the imaging methods**. Computed microscope images (normalized) and the central slices from the EMD and chirped-*z* transform based imaging methods. The field of view for both insets is 4 μm × 4 μm in the sample space. Each pixel on the microscope image is 2 μm × 2 μm, which corresponds to 20 nm × 20 nm in the sample space. The gap for the pair of dipoles is $\lambda/15$.

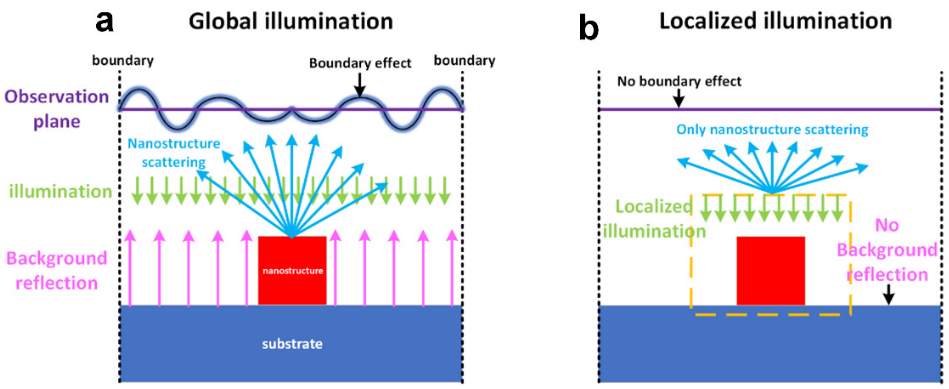

Supplementary Figure 7. **Schematics showing the effect of illumination extent on the observed signals**. **a** Global and **b** localized illumination. The observation plane, scattering from nanostructures, illumination, background reflection, and computation boundary are represented by the purple line, blue arrows, green arrows, lilac arrows, and dashed black lines, respectively. The dashed yellow box in **b** represents the region where localized illumination is applied.

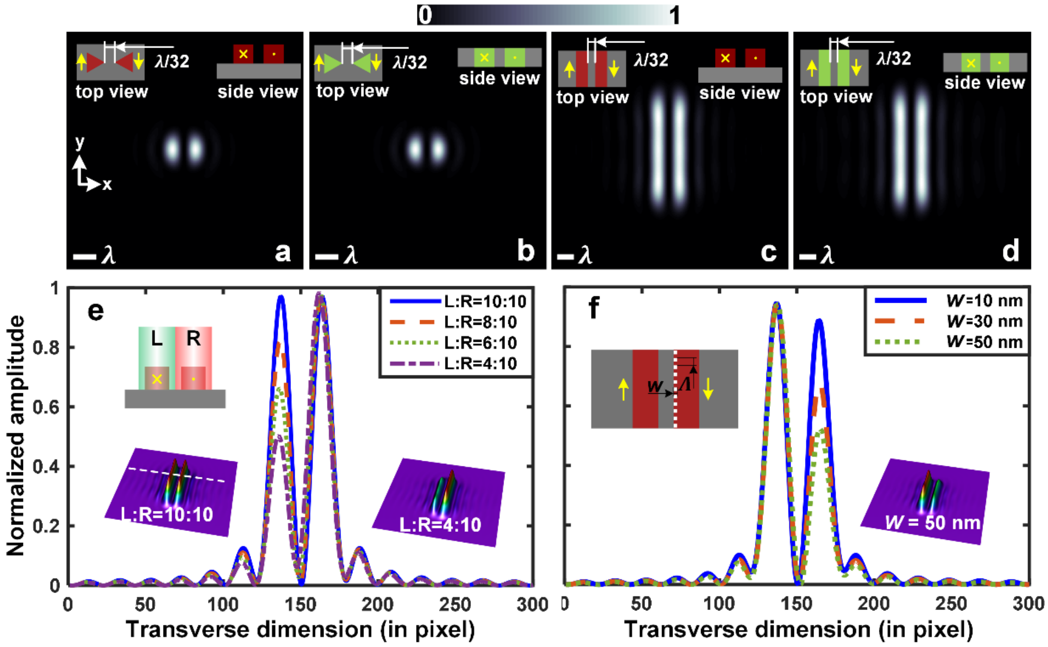

Supplementary Figure 8. **Creation of ECs using anti-symmetric excitation of bowtie and double-nanowire structures**. **a** and **b** Brightfield images of the bowtie in reflection and transmission modes, respectively. **c** and **d** Corresponding images for the double-nanowire structure. The edge-to-edge distance is $\lambda/32$ for all cases, and the side length of the bowtie and the width of the lines are $\lambda/10$ and $\lambda/13$, respectively. The field of view of **a** and **d** is 4 μm × 4 μm in the sample space. **e** and **f** Effect of nonuniform illumination and LER on the quality of the generated EC for the double-nanowire. The central cross-sections for the brightfield images under various right-side amplitudes of **e** excitation strength and **f** LER. L: amplitude of the left-side excitation; R: amplitude of the right-side excitation. w: width of the LER; Λ: pitch of the LER. The gap and duty cycle are fixed at 80 nm and 0.5, respectively. The magnification and input numerical aperture of the brightfield microscope are 100× and 0.8, respectively. Each pixel on the microscope image is 2 μm × 2 μm, which corresponds to 20 nm × 20 nm in the sample space.

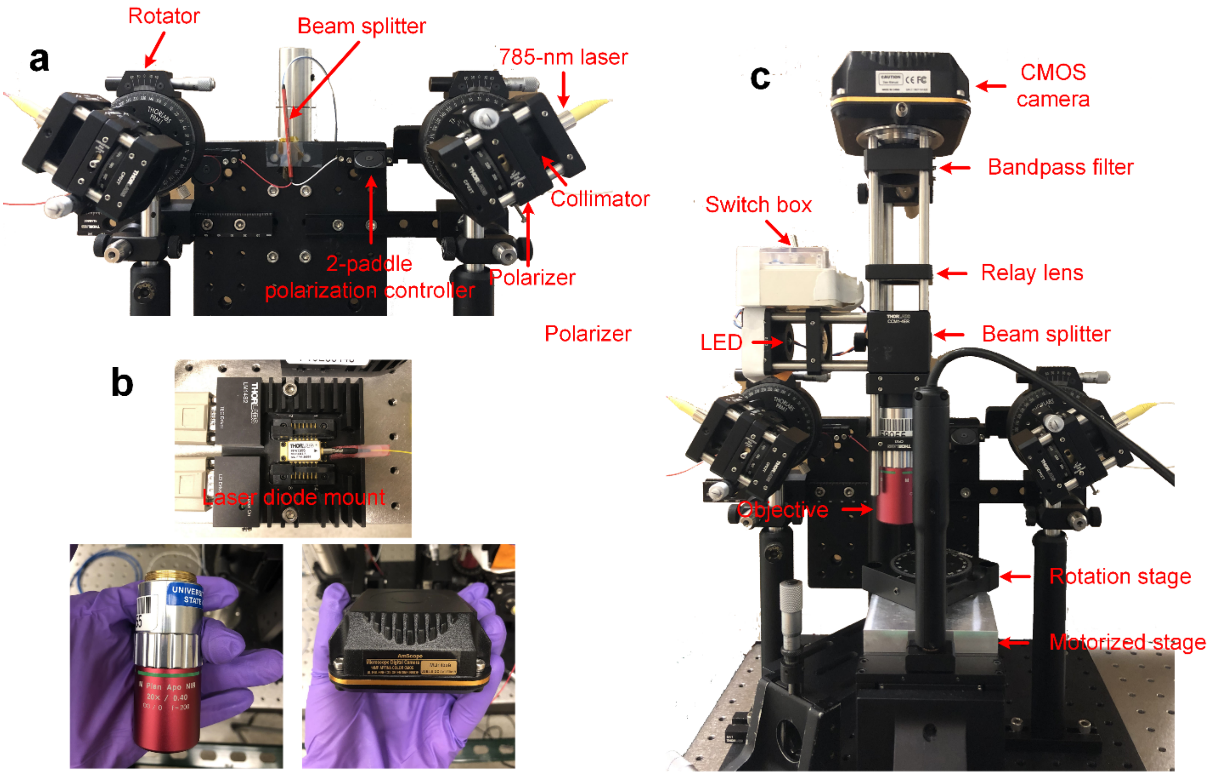

Supplementary Figure 9. Photographs of the experimental systems with primary components marked out. a The two-beam interference apparatus for generating the ECs. **b** The 785-nm laser diode mount, the near infrared (NIR) objective, and the low-cost CMOS camera used in the system. **c** Full view photograph of the entire experimental system including the two-beam interference apparatus and the top-down widefield microscope.

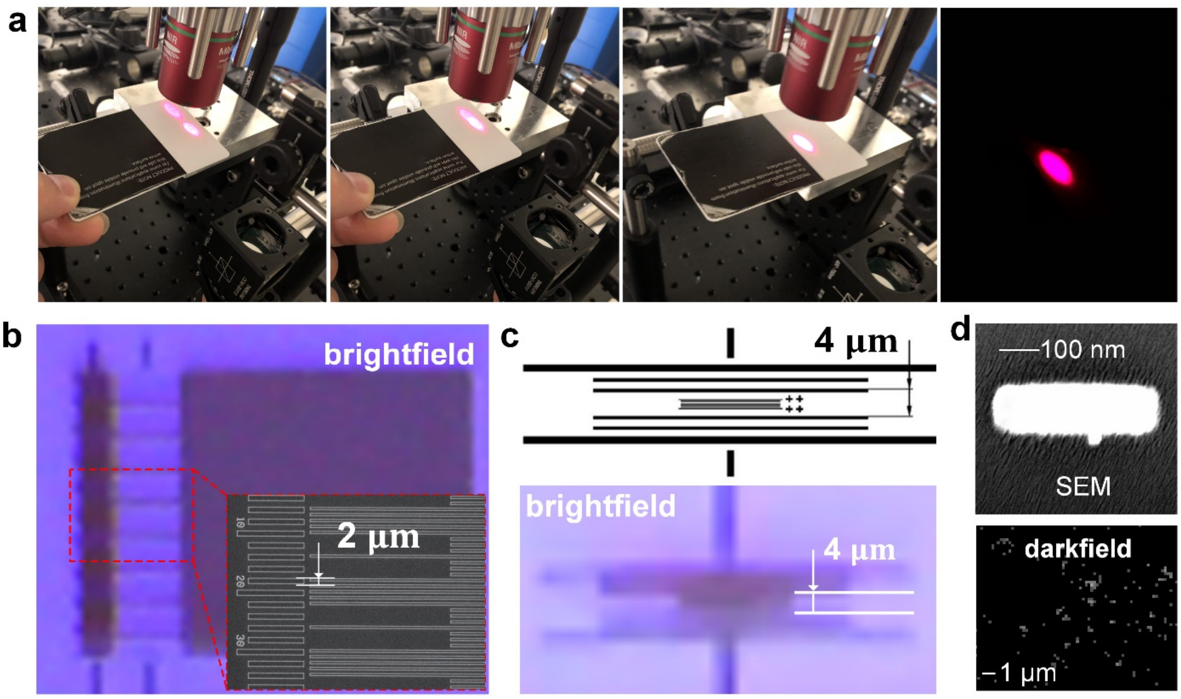

Supplementary Figure 10. **Calibration and testing for the experimental system**. **a** Images during alignment for showing the merging process of the two beams. The merged beam spot with a minimal area taken in a darkened room is shown on the very right panel of **a**. **b** Optical image captured by the top-down widefield microscope under conventional white-light illumination for a region (2-μm pitch; see the inset SEM image adapted from Refs. 11) that consists of parallel lines on the NIST RM8820 artifact. **c** Optical image corresponding to another pattern on the NIST artifact with a 4-μm gap that can be barely observed from the brightfield image. **d** SEM and darkfield images of a nanoparticle. One cannot find the information of the nanoparticle from the darkfield image because of the low performance of the imaging system.

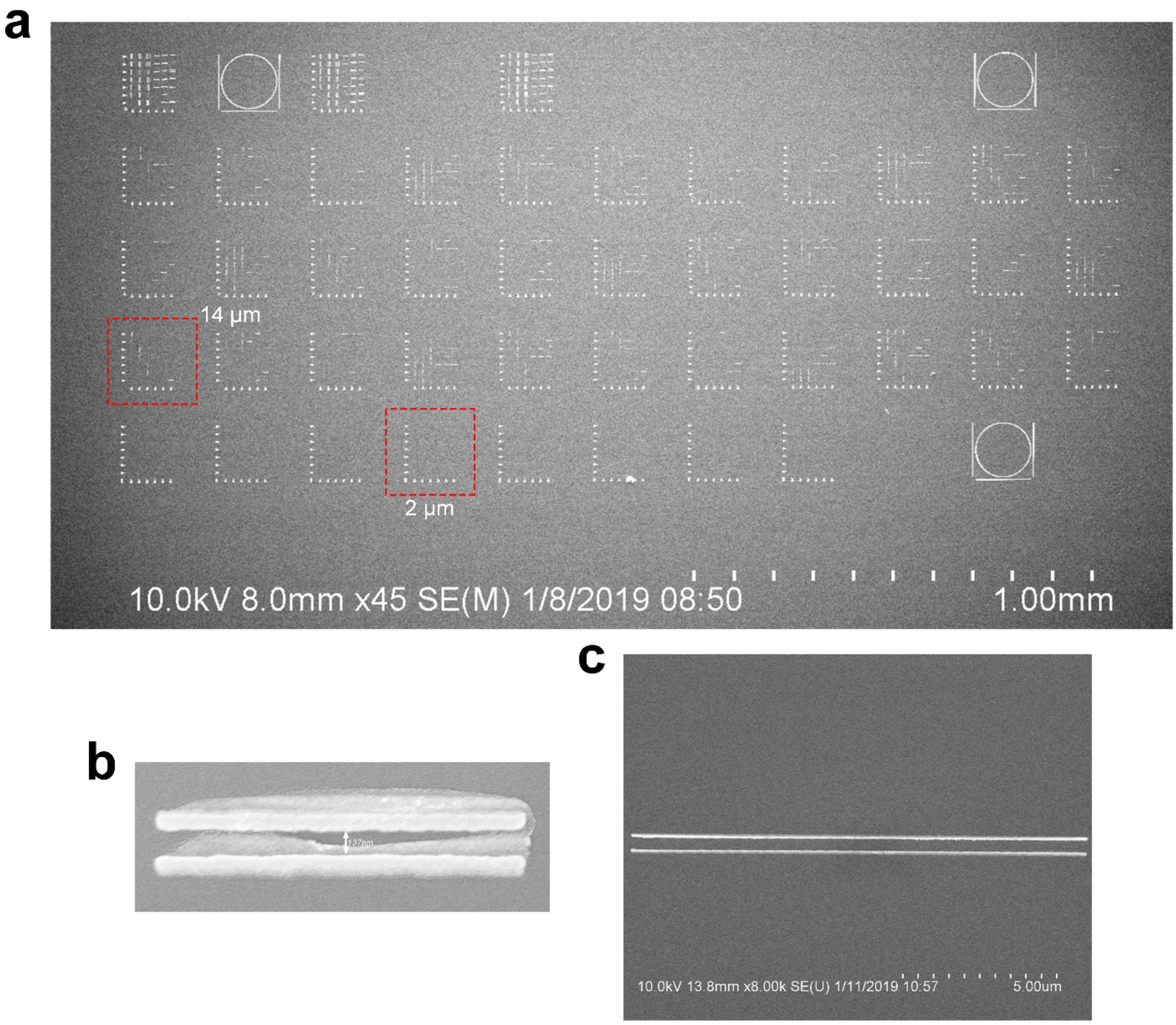

Supplementary Figure 11. **SEM images of the fabricated samples**. **a** A full-view SEM image of the entire set of patterns on the wafer. **b** A representative SEM image of a 2-µm double-nanowire structure that was taken from the die marked by “2 µm” in **a**. **c** A representative SEM image of a typical double-nanowire structure with a nominal length of 14-µm that was taken from the die marked by “14 µm” in **a**.

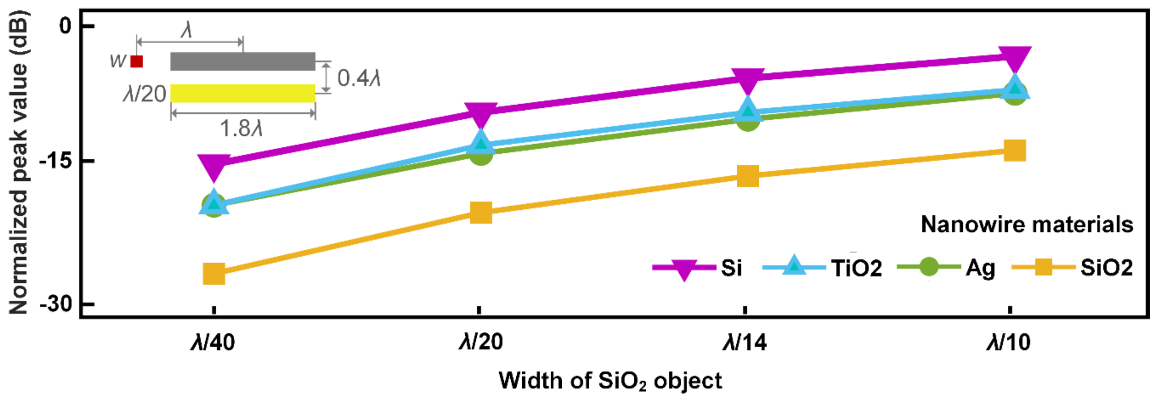

Supplementary Figure 12. **Simulated far-field signals versus the size of a SiO_2 object for different nanowire materials**. Both the length and the width of the object are scaled whereas the size of the nanowires is kept fixed with the dimensions given in the top left corner schematic.

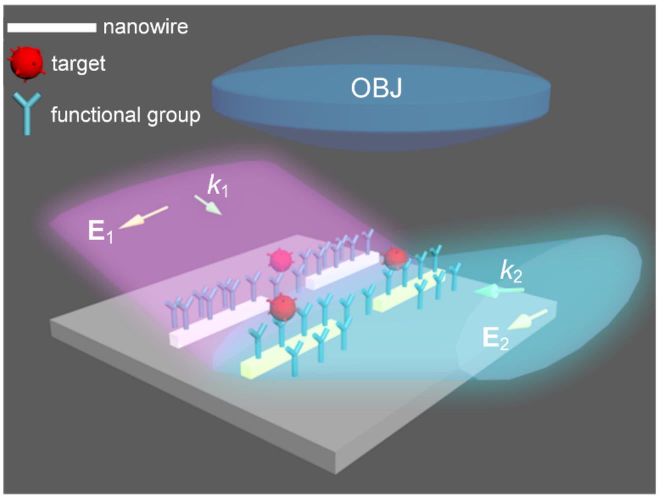

Supplementary Figure 13. The artwork showing the envisioned sensing of biomaterials using the proposed visualization system.

Supplementary Table 1. **Symmetry of the near-field electric field components for different dipole excitations with respect to the planes $x = 0$ and $y = 0$, respectively.**

Excitation	E_x	E_y	E_z
Conventional *x*-polarized	**S, S**	A, A	A, S
Conventional *y*-polarized	A, A	**S, S**	S, A
Conventional *z*-polarized	A, S	S, A	**S, S**
Anisotropic *x*-polarized	**A, S**	S, A	S, S
Anisotropic *y*-polarized	S, A	**A, S**	A, A
Anisotropic *z*-polarized	S, S	A, A	**A, S**

The dominant field component is in bold. (S: symmetric state; A: anti-symmetric state)

Supplementary References

[1] Mayergoyz, I. D., Fredkin, D. R. & Zhang, Z. Electrostatic (plasmon) resonances in nanoparticles. *Phys. Rev. B* **72**, 155412 (2005).

[2] Vernon, K. C., Funston, A. M., Novo, C., Gómez, D. E., Mulvaney, P. & Davis, T. J. Influence of Particle-Substrate Interaction on Localized Plasmon Resonances. *Nano Lett.* **10**, 2080–2086 (2010).

[3] Sobhani, A., Manjavacas, A., Cao, Y., McClain, M. J., Abajo, F. J. G. de, Nordlander, P. & Halas, N. J. Pronounced linewidth narrowing of an aluminum nanoparticle plasmon resonance by interaction with an aluminum metallic film. *Nano Lett.* **15**, 6946-6951 (2015).

[4] Jackson, J. D. *Classical Electrodynamics*. (Wiley, Inc., 1975).

[5] Gu, M. *Advanced Optical Imaging Theory*. (Springer, Inc., 2000).

[6] Gibson, W. C. *The method of moments in electromagnetics*. (Taylor & Francis, Inc., 2015).

[7] Munro, P. R. T. & Torok, P. Calculation of the image of an arbitrary vectorial electromagnetic field. *Opt. Express* **15**, 9293-9307 (2007).

[8] Torok, P., Munro, P. R. T. & Kriezis, E. E. High numerical aperture vectorial imaging in coherent optical microscopes. *Opt. Express* **16**, 507-523 (2008).

[9] Schuck, P. J., Fromm, D. P., Sundaramurthy, A., Kino, G. S. & Moerner, W. E. Improving the mismatch between light and nanoscale objects with gold bowtie nanoantennas. *Phys. Rev. Lett.* **94**, 017402 (2005).

[10] Roxworthy, B. J., Ko, K. D., Kumar, A., Fung, K. H., Chow, E. K. C., Liu, G. L., Fang, N. X. & Toussaint Jr., K. C. Application of plasmonic bowtie nanoantenna arrays for optical trapping, stacking, and sorting. *Nano Lett.* **12**, 796-801 (2012).

[11] Postek, M. T., Vladar, A. E., Keery, W., Bishop, M., Bunday, B. & Allgair, J. Reference Material (RM) 8820: A Versatile New NIST Standard for Nanometrology; Raymond, C. *Proc. SPIE* **76381**, 76381B (2010).
