# Supplementary material for: Visualizable detection of nanoscale objects using anti-symmetric excitation and non-resonance amplification
Source: Nat Commun. 2020 Jun 2;11:2754. doi: 10.1038/s41467-020-16610-0 (PMC7265281; doi:10.1038/s41467-020-16610-0)
Supplement: Supplementary file 3 — Description of Additional Supplementary Files [file 41467_2020_16610_MOESM3_ESM.pdf]

Description of Additional Supplementary Files

File Name: Supplementary Data 1
Description: Archive contains a Graphic Database System (GDS) file that provides the layout of the 3 sample patterns in units of microns and a Portable Document Format (PDF) showing the layout at 1000x magnification.
