# Supplementary material for: Visualizable detection of nanoscale objects using anti-symmetric excitation and non-resonance amplification
Source: Nat Commun. 2020 Jun 2;11:2754. doi: 10.1038/s41467-020-16610-0 (PMC7265281; doi:10.1038/s41467-020-16610-0)

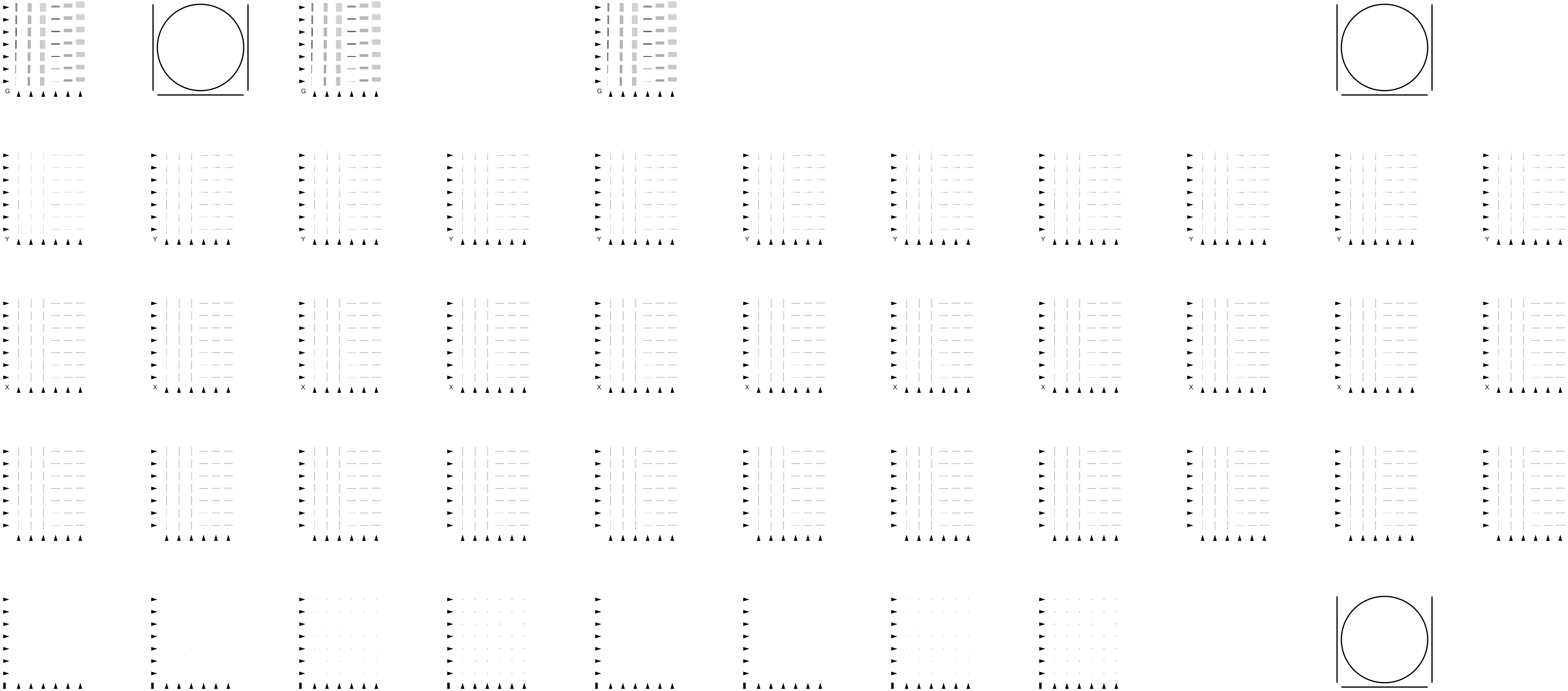

Supplement: Supplementary file 4 — Supplementary Data 1 [file ElectronBeamMaskPattern_1000x_4000dpi.pdf › ElectronBeamMaskPattern_1000x_4000dpi.pdf]
